# Emerging Therapeutic Strategies in Cardiovascular Diseases

**DOI:** 10.7759/cureus.64388

**Published:** 2024-07-12

**Authors:** Rajinderpal Singh, Sohbat Kaur Chandi, Seerat Sran, Smriti K Aulakh, Gurkamal Singh Nijjar, Kanwarmandeep Singh, Sumerjit Singh, FNU Tanvir, Yasmeen Kaur, Ajay Pal Singh Sandhu

**Affiliations:** 1 Internal Medicine, Government Medical College Amritsar, Amritsar, IND; 2 Internal Medicine, Sri Guru Ram Das University of Health Sciences and Research, Amritsar, IND; 3 Medicine, Government Medical College Amritsar, Amritsar, IND; 4 Medicine, Sri Guru Ram Das University of Health Sciences and Research, Amritsar, IND

**Keywords:** rna-based therapies, stem cell therapy, gene therapy, pharmacological treatments, emerging therapies, stroke, ischemic heart disease, emerging treatments, therapeutic strategies, cardiovascular diseases

## Abstract

Cardiovascular diseases (CVDs), including ischemic heart disease and stroke, are the leading cause of mortality worldwide, causing nearly 20 million deaths annually. Traditional therapies, while effective, have not curbed the rising prevalence of CVDs driven by aging populations and lifestyle factors. This review highlights innovative therapeutic strategies that show promise in improving patient outcomes and transforming cardiovascular care. Emerging pharmacological treatments, such as proprotein convertase subtilisin/kexin type 9 (PCSK9) inhibitors and sodium-glucose co-transporter 2 (SGLT2) inhibitors, introduce novel mechanisms to complement existing therapies, significantly reducing cardiovascular events and mortality. These advancements emphasize the necessity of ongoing clinical trials and research to discover new therapeutic targets. Advanced biological therapies, including gene therapy, stem cell therapy, and RNA-based treatments, offer groundbreaking potential for repairing and regenerating damaged cardiovascular tissues. Despite being in various stages of clinical validation, early results are promising, suggesting these therapies could fundamentally change the CVD treatment landscape. Innovative medical devices and technologies, such as implantable devices, minimally invasive procedures, and wearable technology, are revolutionizing CVD management. These advancements facilitate early diagnosis, continuous monitoring, and effective treatment, driving care out of hospitals and into homes, improving patient outcomes and reducing healthcare costs. Personalized medicine, driven by genetic profiling and biomarker identification, allows for tailored therapies that enhance treatment efficacy and minimize adverse effects. However, the adoption of these emerging therapies faces significant challenges, including regulatory hurdles, cost and accessibility issues, and ethical considerations. Addressing these barriers and fostering interdisciplinary collaboration are crucial for accelerating the development and implementation of innovative treatments. Integrating emerging therapeutic strategies in cardiovascular care holds immense potential to transform CVD management. By prioritizing future research and overcoming existing challenges, a new era of personalized, effective, and accessible cardiovascular care can be achieved.

## Introduction and background

Cardiovascular diseases (CVDs), particularly ischemic heart disease and stroke, remain the leading cause of mortality worldwide, accounting for nearly 20 million deaths annually [1]. Three-quarters of cardiovascular-related deaths occur in low- and middle-income settings [2]. This staggering figure underscores the persistent and pervasive burden CVDs impose on global health systems. The economic and social impacts of these diseases are profound, affecting not only individuals and families but also national economies due to the high costs of treatment and lost productivity. Traditional therapies, including lifestyle modifications, pharmacological treatments, and surgical interventions, have significantly improved patient outcomes over the past decades. However, despite these advancements, the prevalence of CVDs continues to rise, driven by factors such as aging populations, increasing rates of obesity, and lifestyle-related risk factors [3].

Given the complexity and multifactorial nature of CVDs, there is a critical need for innovative therapeutic strategies that go beyond the current standard of care. Emerging therapies hold the promise of addressing unmet medical needs, improving patient outcomes, and potentially altering the course of CVDs. These innovative approaches include advanced pharmacological treatments, biological therapies, cutting-edge medical devices, and personalized medicine. Each of these strategies offers unique mechanisms of action and potential benefits, contributing to a more comprehensive and effective management of cardiovascular conditions.

The primary objective of this narrative review is to provide a comprehensive overview of the emerging therapeutic strategies in the treatment of CVDs. By examining the latest advancements in pharmacology, biological therapies, medical devices, and personalized medicine, this review aims to highlight the potential these innovations have in transforming cardiovascular care. The review will also discuss the challenges and future directions in the adoption and implementation of these therapies, considering regulatory, economic, and ethical aspects. Thus, this review endeavors to contribute to the ongoing efforts to reduce the global burden of CVDs and improve cardiovascular health outcomes.

## Review

Methods

In this review article, we summarized current advancements in CVD therapies, focusing on pharmacological, biological, and technological innovations. We conducted a comprehensive literature search across multiple databases, including PubMed, Medline, Embase, and Cochrane Library, targeting studies published from 2013 to 2023. Keywords included "cardiovascular diseases," "innovative therapies," "PCSK9 inhibitors," "SGLT2 inhibitors," "gene therapy," "stem cell therapy," "RNA-based treatments," "medical devices," "wearable technology," and "personalized medicine." Studies were selected based on their relevance to emerging CVD therapies, focusing on randomized controlled trials, observational studies, and meta-analyses. Data were extracted and summarized on new therapeutic mechanisms, clinical efficacy, and stages of clinical validation. We evaluated the impact of these therapies on cardiovascular events, mortality rates, tissue regeneration, and patient outcomes. Additionally, the potential of personalized medicine in CVD treatment was analyzed through studies on genetic profiling and biomarker identification. Challenges to adopting these therapies, including regulatory, cost, and accessibility issues, were examined, along with ethical considerations and the need for interdisciplinary collaboration. Insights from leading experts in cardiology, pharmacology, and biotechnology were incorporated to provide a comprehensive overview of the current state and future directions of CVD therapy. This methodology ensured a thorough examination of the latest advancements, highlighting the transformative potential of innovative therapies in improving patient outcomes and shaping the future of cardiovascular care.

Factors contributing to CVD

Although the exact pathogenic landscape of CVDs remains incompletely understood, atherosclerosis has been identified as a deadly, complex, and multifaceted illness and is considered the main cause of this devastating condition [4, 5]. The deposition of lipids in the coronary arteries and the resulting inflammatory reactions are suggested to be the primary cause of atherosclerosis, which ultimately results in acute myocardial infarction (AMI) [6]. Defective endothelium, lipid-laden macrophages, smooth muscle cells, and T lymphocytes are key factors in the development of atherosclerotic plaque. Pathological remodeling of these components can lead to various cardiovascular complications [6, 7]. Atherosclerosis can cause chronic chest pain (angina) even without complete blockage of the coronary arteries. Its chronic progression can cause heart enlargement or remodeling, resulting in major or minor myocardial infarctions (MIs) and ultimately heart failure. This condition is characterized by symptoms including shortness of breath during exertion and fluid buildup in the legs and lungs. Other mechanisms, such as hypertension and hereditary conditions that impair heart muscle function, are also suggested as potential causes of heart failure. Stroke, another manifestation of cardiovascular pathology, is defined by cerebrovascular occlusions leading to ischemic brain tissue infarction. Approximately 80% of strokes are ischemic, caused by a lack of blood flow to the brain, while 10-20% result from infarcted tissues leading to brain hemorrhages. Thrombosis, embolism, and abnormal coagulation are primary pathophysiological mechanisms linked to stroke [8]. Epidemiological studies suggest that hypertension substantially contributes to CVD [9, 10], and there is evidence of a direct correlation between stroke and coronary heart disease in hypertensive individuals [11]. Hypertension exacerbates endothelial dysfunction by subjecting blood vessels to mechanical stress and increasing intima permeability, crucial in advancing atherosclerosis and plaque rupture [11]. The occurrence and frequency of hypertension rise with age [12]. Unlike other CVDs, hypertension frequently exhibits no symptoms [13], yet increases the risk of significant atherosclerotic events such as stroke, cardiac failure, peripheral arterial disease, and coronary artery disease (CAD) [14]. Understanding these intricate systems could aid in formulating innovative approaches for managing CVD.

**Figure 1:**
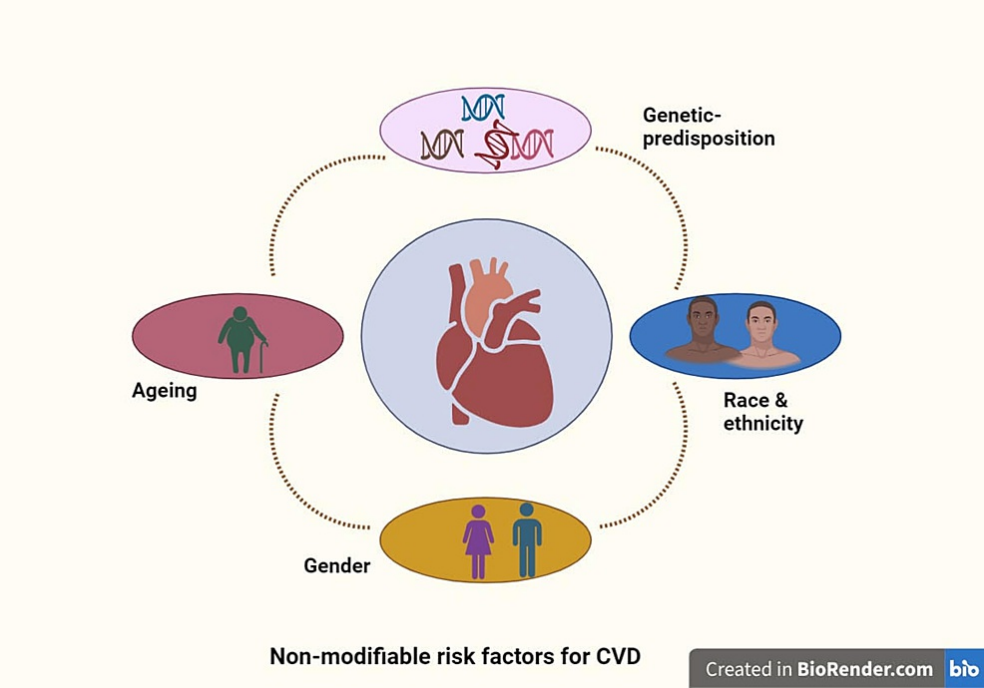
Non-modifiable risk factors for cardiovascular diseases. Created with "BioRender.com".

**Figure 2:**
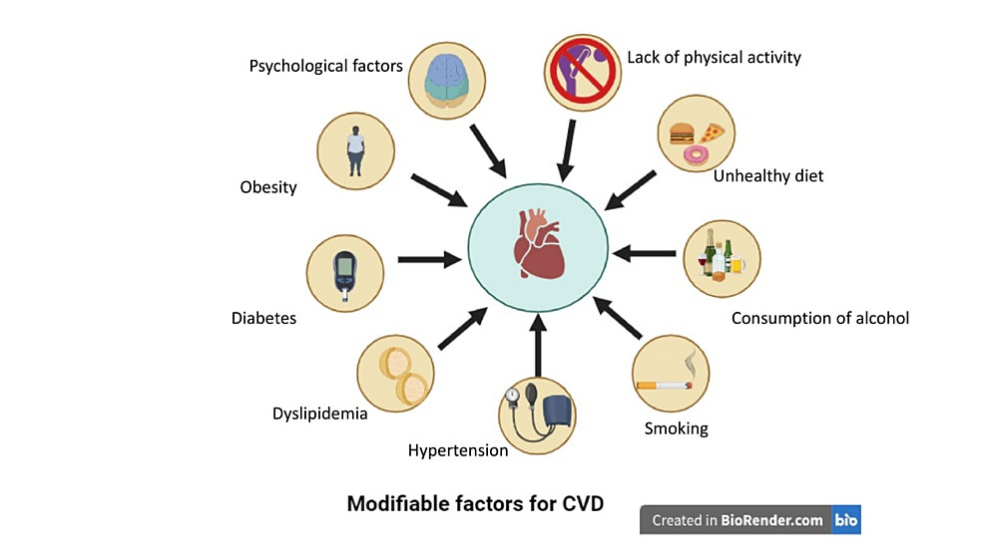
Modifiable risk factors for cardiovascular diseases Created with "BioRender.com".

In addition to the above non-modifiable risk factors, there are modifiable risk factors for CVDs which include smoking, high blood pressure, high cholesterol levels, unhealthy diet, physical inactivity, overweight and obesity, diabetes, excessive alcohol consumption, stress, poor sleep, and social isolation (Figures 1, 2).

Current landscape of cardiovascular therapies

The treatment landscape for CVDs is multifaceted, comprising a combination of pharmacological treatments, surgical interventions, and lifestyle modifications. These existing therapeutic strategies have been pivotal in managing and mitigating the impact of CVDs, contributing to significant reductions in morbidity and mortality over the years [15].

Pharmacological treatments form the cornerstone of CVD management. Statins, widely prescribed for their cholesterol-lowering effects, have been instrumental in reducing the incidence of atherosclerotic CVD [16]. By inhibiting the enzyme HMG-CoA reductase, statins effectively lower low-density lipoprotein (LDL) cholesterol levels, thereby reducing the risk of MI and stroke [17, 18]. Beta-blockers, another critical class of drugs, work by blocking the effects of adrenaline on the heart, slowing the heartbeat, and reducing blood pressure [19]. This makes them invaluable in treating conditions such as hypertension, heart failure, and arrhythmias. Additionally, angiotensin-converting enzyme (ACE) inhibitors and angiotensin II receptor blockers (ARBs) are commonly used to manage hypertension and heart failure, providing benefits by dilating blood vessels and improving blood flow [20]. Table 1 describes various types of drugs, their mode of action, and side effects in the treatment of CVDs.

**Table 1:** Drugs used in cardiovascular diseases, their classification, mode of action, and side effects. HTN: hypertension; HF: heart failure; MI: cardiac infarction; N/V: nausea and vomiting; hypo T: hypothyroidism; HDL: high-density lipoproteins; CAD: coronary artery disease; NVCD: nausea, vomiting, constipation, diarrhea; ASA: acetylsalicylic acid; GI-gastrointestinal; A.fib/flutter-atrial fibrillation flutter; DVT-deep vein thrombosis; PE: pulmonary embolism; HR: heart rate; BP: blood pressure; SVT: supraventricular tachycardia; ARF: acute rheumatic fever; N/V/C: nausea, vomiting, constipation; AV block: atrioventricular block; PR: pulse rate; CHF: congestive heart failure; RVR: rapid ventricular response; VT/VF: ventricular tachycardia/ventricular fibrillation; SOB: shortness of breath; PCSK9 inhibitors: proprotein convertase subtilisin/kexin type 9; SGLT2 inhibitors: sodium-glucose co-transporter 2; NOACs: novel oral anticoagulants.

Classification	Mode of action	Drug name	Cardiac treatment	Side effects
Direct vasodilators	Relax arteriolar smooth muscle, causing blood vessel dilation	Hydrazaline (isosorbide mononitrate); nitroglycerin (sodium nitroprusside)	HTN, chronic stable angina, HF after MI	Headache, dizziness, palpitation, N/V, hypo T, flushing
Statin drugs	Inhibits synthesis of cholesterol in the liver	Atoravastain; lovastatin; simvastatin; fluvastatin	HDL, CAD	NVCD, elevated liver enzymes, myopathy, rhabdomyolysis, GI disturbances, rash
Antiplatelet	Decrease platelet aggregation and inhibit thrombus formation	ASA; clopidogrel bisulfate	MI or re-infarction, CAD, stroke	Hematuria, bruising, epistaxis, confusion, GI ulcers, or upset, hemorrhage
Anticoagulation	Prolong the formation of blood clotting	Warfarin; heparin; enoxaparin	A.fib/flutter, MI, DVT, PE, stroke	HR, BP, bruising, petechiae, black/tarry stools, bleeding in urine/gums, vasculitis, hemorrhage
Ace inhibitors	↓ Conversion of A-I to A-II; vasodilator	Captopril; enalapril; lisinopril; ramipril; trandolapril; fosinapril	HTN, CAD, SVT, A. fib/flutter, junctional dysrhythmia, chronic stable angina	Hypo T, dizziness, ARF, ↑ K^+^, angioedema, skin rash, cough, loss of taste, N/V/C, GI irritation
Beta-blockers	Decrease HR	Atenolol; darvedilol; metoprolol, sotalol	HTN, AV block, SVT, A. fib/flutter, bradycardia, impaired peripheral circulation, stable angina	N/V, Brady, P hypo T, fatigue, bronchospasms, hyperglycemia, head/dizziness, drowsiness, CHF, ED
Ca^+ ^channel blockers	Decrease conduction	Verapamil; diltiazem; amlodipine; nifedipine; felodipine; nicardipine	HTN, A.fib/flutter, SVT, junctional dysrhythmia, chronic stable angina	AV block (prolonged PR interval), bradycardia, hypo T, pulmonary edema, CHF, headache, dizziness, flushing, rash, fever, chills
K^+ ^channel blockers	Slow down action potential (fibrillation)	Amiodarone; propafenone; procainamide; ibutilide, sotalol	A.fibw/RVR, SVT, VT/VF	HF, AV block, pulmonary toxicity, painful breathing, cough, SOB, weakness in arms/legs, trouble walking, dizziness, and lightheadedness
PCSK9 inhibitors	Increases the number of LDL receptors on liver cells leading to increased clearance of LDL cholesterol from the bloodstream	Evolocumab, alirocumab	Lipid management, lowering LDL cholesterol	Injection site reactions
SGLT2 inhibitors	Promote glucose excretion in the urine, induce osmotic diuresis, and reduce preload, and afterload on the heart	Empagliflozin, canagliflozin, dapagliflozin	Reducing cardiovascular events, improving heart failure outcomes	Genital infections, ketoacidosis (in diabetic patients), Rare lower limb amputations
NOACs	Directly inhibit key factors in the coagulation cascade. These drugs have predictable pharmacokinetics, minimal food and drug interactions, quick onset of action, and a relatively short plasma half-life	Dabigatran, rivaroxaban, apixaban, edoxaban	Preventing stroke in atrial fibrillation, treating and preventing venous thromboembolism	Bleeding risk, especially in patients with renal impairment or on certain medications

However, surgical interventions are often necessary for patients with advanced or refractory CVD. Coronary artery bypass grafting (CABG) is a well-established procedure that improves blood flow to the heart muscle by diverting blood around clogged arteries [21]. This surgery is particularly beneficial for patients with severe coronary artery disease. Valve replacements, including both mechanical and biological prostheses, are critical for treating valvular heart diseases such as aortic stenosis and mitral regurgitation [22]. These interventions can significantly improve the quality of life and survival rates in affected patients. Minimally invasive techniques, such as transcatheter aortic valve replacement (TAVR), are also gaining popularity due to their reduced recovery times and lower risk profiles compared to traditional open-heart surgery [23].

Nonetheless, lifestyle modifications are essential components of CVD management and prevention. These measures include dietary changes, regular physical activity, smoking cessation, and weight management [24]. A heart-healthy diet, rich in fruits, vegetables, whole grains, and lean proteins, can help reduce risk factors such as hypertension, hyperlipidemia, and obesity. Regular physical activity, such as aerobic exercises and strength training, improves cardiovascular fitness and overall health. Smoking cessation is crucial, as smoking is a major risk factor for numerous cardiovascular conditions. Comprehensive smoking cessation programs, including counseling and pharmacotherapy, are effective in helping individuals quit. Preventative measures also encompass regular health screenings and the management of comorbid conditions such as diabetes and hyperlipidemia through appropriate medical therapies and lifestyle adjustments.

While these strategies have markedly improved outcomes for many patients, the ongoing burden of CVD underscores the need for continued innovation and refinement in therapeutic approaches.

Emerging pharmacological treatments

The landscape of CVD treatment is rapidly evolving with the development of novel drug classes that offer new mechanisms of action. These emerging pharmacological treatments have the potential to significantly enhance patient outcomes by addressing unmet needs in CVD management.

Novel Drug Classes and Their Mechanisms of Action

PCSK9 inhibitors: Proprotein convertase subtilisin/kexin type 9 (PCSK9) inhibitors are a groundbreaking class of drugs that have revolutionized lipid management [25]. PCSK9 is an enzyme that degrades low-density lipoprotein (LDL) receptors in the liver. By inhibiting PCSK9, these drugs increase the number of LDL receptors available to clear LDL cholesterol from the bloodstream, thereby significantly lowering LDL levels [26]. The monoclonal antibodies evolocumab and alirocumab are prominent examples of PCSK9 inhibitors, demonstrating robust efficacy in reducing cardiovascular events in patients with hypercholesterolemia and those intolerant to statins [27].

SGLT2 inhibitors: Sodium-glucose co-transporter 2 (SGLT2) inhibitors were initially developed to treat type 2 diabetes by promoting glucose excretion in the urine [28]. However, they have shown remarkable benefits in reducing cardiovascular events and improving heart failure outcomes [28]. These drugs, including empagliflozin, canagliflozin, and dapagliflozin, reduce the risk of hospitalization for heart failure and slow the progression of chronic kidney disease [29]. Their mechanisms of action include osmotic diuresis, reduction of preload and afterload on the heart, and potential direct myocardial benefits.

New anticoagulants: The development of novel oral anticoagulants (NOACs) has provided safer and more convenient options for patients requiring anticoagulation. Unlike traditional vitamin K antagonists (e.g., warfarin), NOACs such as dabigatran, rivaroxaban, apixaban, and edoxaban offer predictable pharmacokinetics [30]. These anticoagulants have minimal interaction with food or medications, allowing them to be provided in a set dose without the need for regular surveillance [31]. Additionally, these medications have a quick onset of action, a generally predictable pharmacokinetic profile, and a relatively short plasma half-life. This makes it much easier to start, continue, and stop anticoagulant therapy [31]. These drugs work by directly inhibiting key factors in the coagulation cascade, specifically thrombin (dabigatran) or factor Xa (rivaroxaban, apixaban, edoxaban). They are used to prevent stroke in atrial fibrillation and treat and prevent venous thromboembolism.

Potential Benefits and Risks

The novel pharmacological treatments offer significant benefits but also come with potential risks. PCSK9 inhibitors induce a marked reduction in LDL cholesterol levels and subsequent cardiovascular events, especially beneficial for patients with familial hypercholesterolemia or statin intolerance [26]. However, there is potential for injection site reactions and high cost, which may limit accessibility [32]. While SGLT2 inhibitors improved cardiovascular and renal outcomes, reduced hospitalizations for heart failure, and provided potential weight and blood pressure benefits, there is an increased risk of genital infections, ketoacidosis in patients with diabetes, and, rarely, lower limb amputations [33]. New anticoagulants reduced the risk of stroke and systemic embolism in atrial fibrillation, decreased incidence of venous thromboembolism, and offered ease of use without the need for frequent monitoring. However, these are associated with higher costs compared to warfarin and risks of bleeding, particularly in patients with renal impairment or those on concurrent medications that increase bleeding risk [34].

Clinical Trial Results and Ongoing Research

The efficacy and safety of these novel therapies have been demonstrated in numerous clinical trials. For PCSK9 inhibitors, the FOURIER and ODYSSEY OUTCOMES trials showed significant reductions in cardiovascular events and LDL cholesterol levels, supporting their use in high-risk patients [35, 36]. Similarly, SGLT2 inhibitors have been validated in trials such as EMPA-REG OUTCOME [37], CANVAS [38], and DAPA-HF [39], which highlighted their benefits in reducing cardiovascular death, heart failure hospitalizations, and progression of renal disease.

Research continues to explore the full potential of these drugs. Ongoing studies are investigating the benefits of PCSK9 inhibitors in broader populations, including those without established CVD but with high LDL levels. SGLT2 inhibitors are being studied for their effects on heart failure with preserved ejection fraction (HFpEF), a condition with limited treatment options. Additionally, the exploration of combination therapies, such as SGLT2 inhibitors with other heart failure drugs, aims to optimize patient outcomes [40].

Advanced biological therapies

Gene Therapy

Gene therapy through various mechanisms, including viral vectors, non-viral vectors, and genome editing techniques like CRISPR-Cas9, offers potential advancements in CVD treatments. Targets for gene therapy in CVDs include genes involved in lipid metabolism, inflammation, angiogenesis, and cardiac function regulation.

Gene therapy for CVDs has shown promise in preclinical studies and early-phase clinical trials. For example, targeting the PCSK9 gene using adeno-associated viral vectors has demonstrated significant reductions in LDL cholesterol levels. The existing evidence on PCSK9 editing paves the way for exploring other genetically validated therapy targets in dyslipidemia, such as lipoprotein(a), angiopoietin-like 3, and apolipoprotein CIII. These targets can already be suppressed using antisense oligonucleotides and siRNA [41]. Initial efforts toward achieving these objectives indicate encouraging results [42, 43].

Similarly, gene therapy approaches targeting the vascular endothelial growth factor (VEGF) family have shown potential for promoting angiogenesis in ischemic heart disease [44]. Preclinical investigations conducted on large animals have unequivocally shown that VEGF gene therapy is both safe and effective in disease models that are clinically relevant. Nevertheless, initial clinical trials using the intravascular administration of VEGF vector constructs have yielded only modest advantages for the patients [45]. Second-generation VEGF-based gene therapy experiments utilize direct intramyocardial and intraskeletal muscle injections to enhance transfection efficiency and achieve more precise effects. Currently, Phase I/II studies are underway to assess the safety, feasibility, and efficacy of these enhanced methods in patients suffering from serious cardiovascular illnesses.

However, challenges remain, including the need for more efficient gene delivery systems, concerns about off-target effects and immunogenicity, and long-term safety and efficacy assessments.

Stem Cell Therapy

**Figure 3:**
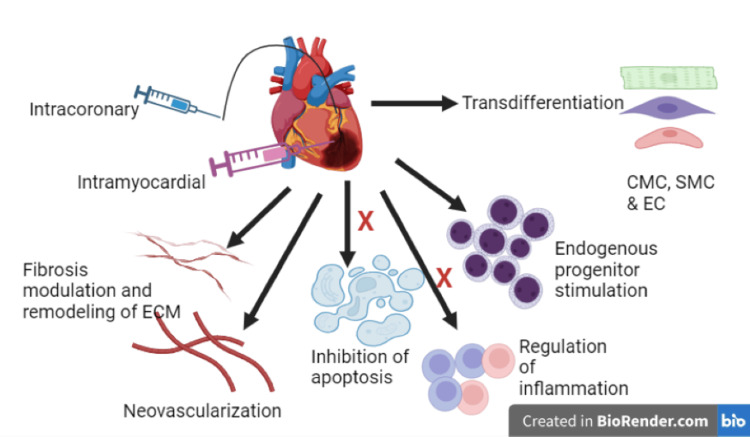
Stem cell therapy for cardiovascular diseases Clinically evaluated and emerging stem and progenitor cell populations, along with their delivery methods for treating heart failure. CMC: cardiac muscle cells; SMC: smooth muscle cells; EC: embryonic cells.

Various types of stem cells have been investigated for their potential in CVD treatment, including embryonic stem cells, induced pluripotent stem cells, mesenchymal stem cells (MSCs), and cardiac progenitor cells (Figure 3). Clinical trials evaluating stem cell therapy in CVDs have yielded mixed results [46, 47]. While some studies have reported improvements in cardiac function, myocardial perfusion, and exercise capacity [48], others have shown limited or transient benefits [49]. Mesenchymal stem cells (MSCs), in particular, have shown promise due to their immunomodulatory properties and ability to differentiate into multiple cell types [50]. However, challenges such as low engraftment rates, cell survival, and potential tumorigenicity need to be addressed to enhance the therapeutic efficacy of stem cell therapy.

RNA-Based Therapies

RNA-based therapies, such as siRNA and mRNA, utilize small interfering RNA (siRNA) or messenger RNA (mRNA) molecules to modulate gene expression and protein production. siRNA targets specific mRNA molecules for degradation, thereby silencing gene expression, while mRNA-based therapies deliver exogenous mRNA to encode therapeutic proteins. In CVDs, RNA-based therapies can target genes involved in lipid metabolism, inflammation, fibrosis, and cardiac remodeling [51].

RNA-based therapies hold great promise for precision medicine in CVDs. Recent advancements in delivery technologies, such as lipid nanoparticles and viral vectors, have improved the stability and specificity of RNA molecules [52]. Clinical trials investigating siRNA-based therapies targeting genes like PCSK9 have shown significant reductions in LDL cholesterol levels [53]. Similarly, mRNA-based vaccines have demonstrated efficacy in preventing cardiovascular events by targeting atherosclerotic plaque formation [54, 55]. However, challenges such as off-target effects, immunogenicity, and scalability need to be addressed to facilitate the clinical translation of RNA-based therapies in cardiovascular medicine [56].

Innovative medical devices and technologies

Implantable Devices

Left ventricular assist devices (LVADs): LVADs are mechanical pumps implanted in patients with advanced heart failure to help the heart pump blood to the rest of the body. These devices can be used as a bridge to transplant, destination therapy for patients ineligible for heart transplantation, or as a bridge to recovery [57]. LVADs have evolved significantly over the years, with improvements in device design, durability, and miniaturization [58]. They have revolutionized the management of end-stage heart failure and significantly improved patient outcomes and quality of life.

Implantable cardioverter-defibrillators (ICDs): ICDs are electronic devices implanted in patients at risk of sudden cardiac death due to life-threatening ventricular arrhythmias. These devices continuously monitor the heart's rhythm and deliver electrical shocks to restore normal heart rhythm if a dangerous arrhythmia is detected [59]. Modern ICDs are equipped with advanced features such as dual-chamber pacing, anti-tachycardia pacing, and remote monitoring capabilities, enhancing their effectiveness and safety in preventing sudden cardiac death [60].

Minimally Invasive Procedures

Transcatheter aortic valve replacement (TAVR): TAVR is a minimally invasive procedure used to treat severe aortic stenosis in patients deemed high risk or inoperable for traditional surgical valve replacement. During TAVR, a collapsible valve is delivered through a catheter and deployed within the native aortic valve, restoring normal blood flow without the need for open-heart surgery [61]. TAVR has rapidly emerged as a viable alternative to surgical aortic valve replacement, offering shorter recovery times, and reduced morbidity and mortality rates [23].

Catheter-based ablation techniques: Catheter-based ablation procedures are minimally invasive interventions used to treat cardiac arrhythmias, including atrial fibrillation, atrial flutter, and ventricular tachycardia. During ablation, catheters with electrodes are inserted into the heart's chambers to deliver radiofrequency or cryotherapy energy to destroy abnormal electrical pathways causing arrhythmias [62]. Advances in catheter technology, imaging guidance, and mapping systems have improved the precision and success rates of ablation procedures, making them an effective treatment option for rhythm disorders [63, 64].

Wearable Technology and Remote Monitoring

Smartwatches and fitness trackers: Wearable devices such as smartwatches and fitness trackers have gained popularity for monitoring various physiological parameters, including heart rate, activity levels, and sleep patterns. These devices provide users with real-time feedback on their health and fitness metrics, enabling early detection of abnormalities and promoting proactive management of cardiovascular risk factors [65, 66].

Telemedicine and remote patient management: Telemedicine platforms and remote monitoring systems allow healthcare providers to remotely monitor patients' vital signs, medication adherence, and disease progression outside of traditional clinical settings. These technologies enable timely intervention, personalized treatment adjustments, and improved patient outcomes, particularly for individuals with chronic cardiovascular conditions or those living in remote areas. Telemedicine also facilitates virtual consultations, empowering patients to access specialized care remotely and reducing the burden on healthcare resources [67].

Personalized medicine in cardiovascular care

Personalized medicine is revolutionizing cardiovascular care by shifting from a one-size-fits-all approach to tailored therapies based on individual patient profiles. This paradigm leverages genetic profiling and biomarkers to enhance the precision and efficacy of treatments for CVDs.

Role of Genetic Profiling and Biomarkers

Genetic profiling involves analyzing a patient’s DNA to identify genetic variations that influence their risk of developing CVDs and their response to various treatments. For instance, variations in genes such as PCSK9 and LDLR are linked to cholesterol levels and cardiovascular risk, guiding the use of statins and other lipid-lowering therapies. Biomarkers, including high-sensitivity C-reactive protein (hs-CRP) and troponins, provide critical insights into the inflammatory and injury status of the cardiovascular system [68]. These markers help in diagnosing conditions like MI and in predicting future cardiovascular events, thereby enabling timely and appropriate therapeutic interventions [69, 70].

Tailoring Therapies Based on Individual Patient Profiles

The integration of genetic and biomarker data allows for the customization of treatment plans. For example, patients with specific genetic mutations that predispose them to higher cholesterol levels may benefit from PCSK9 inhibitors rather than traditional statins [71]. Similarly, antiplatelet therapy can be personalized using genetic information related to the CYP2C19 gene, which affects the metabolism of drugs like clopidogrel [72]. This precision in treatment helps maximize efficacy while minimizing adverse effects. Furthermore, pharmacogenomics can identify patients who are likely to experience side effects from certain medications, allowing for alternative treatments to be considered.

Challenges and future directions

The adoption of emerging therapies in cardiovascular care faces several significant barriers. Regulatory challenges, cost and accessibility issues, and ethical considerations are among the primary obstacles hindering widespread implementation.

One of the most significant barriers is navigating the complex regulatory landscape. Emerging therapies often require rigorous testing and approval processes to ensure safety and efficacy [73]. This can lead to delays in bringing innovative treatments to market. Additionally, differences in regulatory standards across countries can complicate global implementation. Regulatory agencies need to balance the need for thorough evaluation with the urgency of providing patients with access to potentially life-saving treatments.

The high cost of developing and implementing new therapies poses another significant barrier. Many emerging treatments, particularly those involving advanced technologies or personalized medicine approaches, are expensive to produce and administer. This can limit accessibility, especially in low- and middle-income countries. Health insurance coverage for these therapies can also be inconsistent, further restricting patient access [74]. Addressing these cost issues requires coordinated efforts to reduce production costs, increase funding for innovative therapies, and ensure equitable access to all patients, regardless of economic status [75].

Emerging therapies raise various ethical concerns, particularly related to patient consent, privacy, and equity. Genetic profiling and personalized medicine approaches, for instance, involve the collection and analysis of sensitive genetic data, which must be handled with strict confidentiality and informed consent protocols. Additionally, ensuring that new treatments are equitably distributed and do not exacerbate existing healthcare disparities is a critical ethical challenge.

Future research in cardiovascular therapies should focus on several key areas. There is a need for more extensive clinical trials to validate the efficacy and safety of emerging treatments across diverse patient populations. Understanding the long-term effects of novel therapies, particularly those involving genetic and cellular modifications, is crucial. Additionally, research should explore the integration of various therapeutic approaches to optimize patient outcomes.

Interdisciplinary collaboration is essential for advancing cardiovascular therapies. Collaboration between cardiologists, geneticists, bioengineers, and data scientists can drive the development of innovative treatments. Integrating insights from different fields can lead to more comprehensive and effective therapeutic strategies. For instance, combining expertise in molecular biology and materials science could result in more advanced drug delivery systems or implantable devices.

## Conclusions

The landscape of CVD treatment is rapidly evolving, with emerging therapeutic strategies offering promising avenues to improve patient outcomes and quality of life. This review underscores the importance of personalized medicine, advanced biological therapies, novel pharmacological treatments, and cutting-edge medical devices in revolutionizing cardiovascular care. Personalized medicine, driven by genetic profiling and biomarker identification, allows for tailored therapies that address individual patient needs, enhancing treatment efficacy and minimizing adverse effects. Despite challenges in integrating genetic data into clinical practice, the potential benefits emphasize the need for continued research and development.

Advanced biological therapies, including gene therapy, stem cell therapy, and RNA-based treatments, represent groundbreaking advancements with the potential to repair and regenerate damaged cardiovascular tissues. While still in various stages of clinical validation, early results are promising, suggesting a fundamental change in the treatment landscape for CVDs. Novel pharmacological treatments, such as PCSK9 inhibitors and SGLT2 inhibitors, offer new mechanisms of action that complement existing therapies. These drugs have shown significant efficacy in reducing cardiovascular events and mortality, highlighting the importance of ongoing clinical trials and research in identifying new therapeutic targets. Innovative medical devices and technologies, including implantable devices, minimally invasive procedures, and wearable technology, are transforming the management of CVDs. These advancements facilitate early diagnosis, continuous monitoring, and effective treatment, thereby improving patient outcomes and reducing healthcare costs.

Integrating emerging therapeutic strategies in cardiovascular care holds immense potential to transform CVD management. By addressing current challenges and prioritizing future research, we can pave the way for a new era of personalized, effective, and accessible cardiovascular care. Future research should focus on overcoming these barriers and fostering interdisciplinary collaboration to accelerate the development and implementation of innovative treatments.

## References

[1] Di Cesare M, Perel P, Taylor S (2024). The heart of the world. Glob Heart.

[2] Gaziano TA, Bitton A, Anand S, Abrahams-Gessel S, Murphy A (2010). Growing epidemic of coronary heart disease in low- and middle-income countries. Curr Probl Cardiol.

[3] Roth GA, Mensah GA, Johnson CO (2020). Global burden of cardiovascular diseases and risk factors, 1990-2019: update from the GBD 2019 study. J Am Coll Cardiol.

[4] Firoz CK, Jabir NR, Khan MS (2015). An overview on the correlation of neurological disorders with cardiovascular disease. Saudi J Biol Sci.

[5] Perk J, De Backer G, Gohlke H (2012). European guidelines on cardiovascular disease prevention in clinical practice (version 2012). The fifth Joint Task Force of the European Society of Cardiology and other societies on cardiovascular disease prevention in clinical practice (constituted by representatives of nine societies and by invited experts). Eur Heart J.

[6] Gutierrez-Pajares JL, Iturrieta J, Dulam V (2015). Caveolin-3 promotes a vascular smooth muscle contractile phenotype. Front Cardiovasc Med.

[7] Robinson WF, Robinson NA (10.1016/B978-0-7020-5319-1.00012-8). Cardiovascular system. Jubb, Kennedy & Palmer's Pathology of Domestic Animals.

[8] Sughrue T, Swiernik MA, Huang Y, Brody JP (2016). Laboratory tests as short-term correlates of stroke. BMC Neurol.

[9] Picariello C, Lazzeri C, Attanà P, Chiostri M, Gensini GF, Valente S (2011). The impact of hypertension on patients with acute coronary syndromes. Int J Hypertens.

[10] Pedrinelli R, Ballo P, Fiorentini C (2012). Hypertension and acute myocardial infarction: an overview. J Cardiovasc Med (Hagerstown).

[11] Reinstadler SJ, Stiermaier T, Eitel C (2016). Antecedent hypertension and myocardial injury in patients with reperfused ST-elevation myocardial infarction. J Cardiovasc Magn Reson.

[12] Yoon SS, Gu Q, Nwankwo T, Wright JD, Hong Y, Burt V (2015). Trends in blood pressure among adults with hypertension: United States, 2003 to 2012. Hypertension.

[13] Weisfeldt ML, Zieman SJ (2007). Advances in the prevention and treatment of cardiovascular disease. Health Aff (Millwood).

[14] Ciruzzi M, Pramparo P, Rozlosnik J (2001). Hypertension and the risk of acute myocardial infarction in Argentina. The Argentine Factores de Riesgo Coronario en America del Sur (FRICAS) investigators. Prev Cardiol.

[15] Maron DJ, Fazio S, Linton MF (2000). Current perspectives on statins. Circulation.

[16] Liu JR, Liu BW, Yin FZ (2017). Change in nonhigh-density lipoprotein cholesterol levels in adults with prediabetes. Medicine (Baltimore).

[17] Stancu C, Sima A (2001). Statins: mechanism of action and effects. J Cell Mol Med.

[18] Schoenhagen P, Ziada KM, Kapadia SR, Crowe TD, Nissen SE, Tuzcu EM (2000). Extent and direction of arterial remodeling in stable versus unstable coronary syndromes: an intravascular ultrasound study. Circulation.

[19] Frishman WH (2003). Cardiology patient page. Beta-adrenergic blockers. Circulation.

[20] Gao M, Lin W, Ma T, Luo Y, Xie H, Cheng X, Bai Y (2022). The impact of different antihypertensive drugs on cardiovascular risk in isolated systolic hypertension with type 2 diabetes patients. J Clin Med.

[21] Harris R, Croce B, Tian DH (2013). Coronary artery bypass grafting. Ann Cardiothorac Surg.

[22] Taghizadeh B, Ghavami L, Derakhshankhah H (2020). Biomaterials in valvular heart diseases. Front Bioeng Biotechnol.

[23] Srinivasan A, Wong F, Wang B (2024). Transcatheter aortic valve replacement: past, present, and future. Clin Cardiol.

[24] Brinks J, Fowler A, Franklin BA, Dulai J (2017). Lifestyle modification in secondary prevention: beyond pharmacotherapy. Am J Lifestyle Med.

[25] Kim EJ, Wierzbicki AS (2020). The history of proprotein convertase subtilisin kexin-9 inhibitors and their role in the treatment of cardiovascular disease. Ther Adv Chronic Dis.

[26] Hajar R (2019). PCSK 9 inhibitors: a short history and a new era of lipid-lowering therapy. Heart Views.

[27] Ghasempour G, Zamani-Garmsiri F, Shaikhnia F (2024). Efficacy and safety of alirocumab and evolocumab as proprotein convertase subtilisin/kexin type 9 (PCSK9) inhibitors in familial hypercholesterolemia: a systematic review and meta-analysis. Curr Med Chem.

[28] Hasan I, Rashid T, Jaikaransingh V, Heilig C, Abdel-Rahman EM, Awad AS (2024). SGLT2 inhibitors: beyond glycemic control. J Clin Transl Endocrinol.

[29] Kansara A, Mubeen F, Shakil J (2022). SGLT2 inhibitors in patients with chronic kidney disease and heart disease: a literature review. Methodist Debakey Cardiovasc J.

[30] Ciurus T, Sobczak S, Cichocka-Radwan A, Lelonek M (2015). New oral anticoagulants - a practical guide. Kardiochir Torakochirurgia Pol.

[31] Heidbuchel H, Verhamme P, Alings M (2013). European Heart Rhythm Association practical guide on the use of new oral anticoagulants in patients with non-valvular atrial fibrillation. Europace.

[32] Chaudhary R, Garg J, Shah N, Sumner A (2017). PCSK9 inhibitors: a new era of lipid lowering therapy. World J Cardiol.

[33] Yau K, Dharia A, Alrowiyti I, Cherney DZ (2022). Prescribing SGLT2 inhibitors in patients with CKD: expanding indications and practical considerations. Kidney Int Rep.

[34] Vinogradova Y, Coupland C, Hill T, Hippisley-Cox J (2018). Risks and benefits of direct oral anticoagulants versus warfarin in a real world setting: cohort study in primary care. BMJ.

[35] Bittner VA, Szarek M, Aylward PE (2020). Effect of alirocumab on lipoprotein(a) and cardiovascular risk after acute coronary syndrome. J Am Coll Cardiol.

[36] Schwartz GG, Steg PG, Szarek M (2020). Peripheral artery disease and venous thromboembolic events after acute coronary syndrome: role of lipoprotein(a) and modification by alirocumab: prespecified analysis of the Odyssey outcomes randomized clinical trial. Circulation.

[37] Zinman B, Wanner C, Lachin JM (2015). Empagliflozin, cardiovascular outcomes, and mortality in type 2 diabetes. N Engl J Med.

[38] Shah SR, Najim NI, Abbasi Z (2018). Canagliflozin and cardiovascular disease - results of the CANVAS trial. J Community Hosp Intern Med Perspect.

[39] McMurray JJ, DeMets DL, Inzucchi SE (2019). A trial to evaluate the effect of the sodium-glucose co-transporter 2 inhibitor dapagliflozin on morbidity and mortality in patients with heart failure and reduced left ventricular ejection fraction (DAPA-HF). Eur J Heart Fail.

[40] Fatima A, Rasool S, Devi S (2023). Exploring the cardiovascular benefits of sodium-glucose cotransporter-2 (SGLT2) inhibitors: expanding horizons beyond diabetes management. Cureus.

[41] Katzmann JL, Packard CJ, Chapman MJ, Katzmann I, Laufs U (2020). Targeting RNA with antisense oligonucleotides and small interfering RNA: JACC state-of-the-art review. J Am Coll Cardiol.

[42] Chadwick AC, Musunuru K (2018). CRISPR-Cas9 genome editing for treatment of atherogenic dyslipidemia. Arterioscler Thromb Vasc Biol.

[43] Chadwick AC, Evitt NH, Lv W, Musunuru K (2018). Reduced blood lipid levels with in vivo CRISPR-Cas9 base editing of ANGPTL3. Circulation.

[44] Zhou Y, Zhu X, Cui H, Shi J, Yuan G, Shi S, Hu Y (2021). The role of the VEGF family in coronary heart disease. Front Cardiovasc Med.

[45] Niu G, Chen X (2010). Vascular endothelial growth factor as an anti-angiogenic target for cancer therapy. Curr Drug Targets.

[46] Terashvili M, Bosnjak ZJ (2019). Stem cell therapies in cardiovascular disease. J Cardiothorac Vasc Anesth.

[47] Malliaras K, Makkar RR, Smith RR (2014). Intracoronary cardiosphere-derived cells after myocardial infarction: evidence of therapeutic regeneration in the final 1-year results of the CADUCEUS trial (CArdiosphere-Derived aUtologous stem CElls to reverse ventricUlar dySfunction). J Am Coll Cardiol.

[48] Jayawardena TM, Egemnazarov B, Finch EA (2012). MicroRNA-mediated in vitro and in vivo direct reprogramming of cardiac fibroblasts to cardiomyocytes. Circ Res.

[49] Quyyumi AA, Vasquez A, Kereiakes DJ (2017). PreSERVE-AMI: a randomized, double-blind, placebo-controlled clinical trial of intracoronary administration of autologous CD34+ cells in patients with left ventricular dysfunction post STEMI. Circ Res.

[50] Arslan F, Lai RC, Smeets MB (2013). Mesenchymal stem cell-derived exosomes increase ATP levels, decrease oxidative stress and activate PI3K/Akt pathway to enhance myocardial viability and prevent adverse remodeling after myocardial ischemia/reperfusion injury. Stem Cell Res.

[51] Laggerbauer B, Engelhardt S (2022). MicroRNAs as therapeutic targets in cardiovascular disease. J Clin Invest.

[52] Yang L, Gong L, Wang P (2022). Recent advances in lipid nanoparticles for delivery of mRNA. Pharmaceutics.

[53] Katzmann JL, Cupido AJ, Laufs U (2022). Gene therapy targeting PCSK9. Metabolites.

[54] Iversen PL, Kipshidze N, Kipshidze N, Dangas G, Ramacciotti E, Kakabadze Z, Fareed J (2023). A novel therapeutic vaccine targeting the soluble TNFα receptor II to limit the progression of cardiovascular disease: AtheroVax™. Front Cardiovasc Med.

[55] Chyu KY, Dimayuga PC, Shah PK (2017). Vaccine against arteriosclerosis: an update. Ther Adv Vaccines.

[56] Aderinto N, Abdulbasit MO, Olatunji G, Edun M, Aboderin G (2023). The promise of RNA-based therapeutics in revolutionizing heart failure management - a narrative review of current evidence. Ann Med Surg (Lond).

[57] Garbade J, Bittner HB, Barten MJ, Mohr FW (2011). Current trends in implantable left ventricular assist devices. Cardiol Res Pract.

[58] Kubrusly LF (2019). Ventricular assist devices: an evolving field. Braz J Cardiovasc Surg.

[59] Miller MA, Neuzil P, Dukkipati SR, Reddy VY (2015). Leadless cardiac pacemakers: back to the future. J Am Coll Cardiol.

[60] Sahu P, Acharya S, Totade M (2023). Evolution of pacemakers and implantable cardioverter defibrillators (ICDs) in cardiology. Cureus.

[61] Bianchi M, Marom G, Ghosh RP, Fernandez HA, Taylor JR Jr, Slepian MJ, Bluestein D (2016). Effect of balloon-expandable transcatheter aortic valve replacement positioning: a patient-specific numerical model. Artif Organs.

[62] Ghzally Y, Ahmed I, Gerasimon G (2024). Catheter ablation. StatPearls [Internet].

[63] Nof E, Stevenson WG, John RM (2013). Catheter ablation for ventricular arrhythmias. Arrhythm Electrophysiol Rev.

[64] D'Silva A, Wright M (2011). Advances in imaging for atrial fibrillation ablation. Radiol Res Pract.

[65] Masoumian Hosseini M, Masoumian Hosseini ST, Qayumi K, Hosseinzadeh S, Sajadi Tabar SS (2023). Smartwatches in healthcare medicine: assistance and monitoring; a scoping review. BMC Med Inform Decis Mak.

[66] Moshawrab M, Adda M, Bouzouane A, Ibrahim H, Raad A (2023). Smart wearables for the detection of cardiovascular diseases: a systematic literature review. Sensors (Basel).

[67] Haleem A, Javaid M, Singh RP, Suman R (2021). Telemedicine for healthcare: capabilities, features, barriers, and applications. Sens Int.

[68] Wang J, Tan GJ, Han LN, Bai YY, He M, Liu HB (2017). Novel biomarkers for cardiovascular risk prediction. J Geriatr Cardiol.

[69] Korley FK, Jaffe AS (2013). Preparing the United States for high-sensitivity cardiac troponin assays. J Am Coll Cardiol.

[70] Haaf P, Drexler B, Reichlin T (2012). High-sensitivity cardiac troponin in the distinction of acute myocardial infarction from acute cardiac noncoronary artery disease. Circulation.

[71] Beltran RA, Zemeir KJ, Kimberling CR, Kneer MS, Mifflin MD, Broderick TL (2022). Is a PCSK9 inhibitor right for your patient? A review of treatment data for individualized therapy. Int J Environ Res Public Health.

[72] Dean L, Kane M (2012). Clopidogrel therapy and CYP2C19 genotype. Medical Genetics Summaries.

[73] Kepplinger EE (2015). FDA’s expedited approval mechanisms for new drug products. Biotechnol Law Rep.

[74] Allen EM, Call KT, Beebe TJ, McAlpine DD, Johnson PJ (2017). Barriers to care and health care utilization among the publicly insured. Med Care.

[75] Williams JS, Walker RJ, Egede LE (2016). Achieving equity in an evolving healthcare system: opportunities and challenges. Am J Med Sci.

